# The horizontal and vertical components of nystagmus evoked by the supine roll test in horizontal semicircular canal canalolithiasis

**DOI:** 10.3389/fnins.2022.957617

**Published:** 2022-08-24

**Authors:** Xueqing Zhang, Qiaomei Deng, Qiang Liu, Chao Wen, Wei Wang, Taisheng Chen

**Affiliations:** ^1^Department of Otorhinolaryngology Head and Neck Surgery, Tianjin First Central Hospital, Tianjin, China; ^2^Institute of Otolaryngology of Tianjin, Tianjin, China; ^3^Key Laboratory of Auditory Speech and Balance Medicine, Tianjin, China; ^4^Key Medical Discipline of Tianjin (Otolaryngology), Tianjin, China; ^5^Quality Control Centre of Otolaryngology, Tianjin, China

**Keywords:** Ewald’s laws, horizontal semicircular canal, canalolithiasis, video nystagmography, vertical component, BPPV

## Abstract

**Objective:**

The characteristics of horizontal and vertical components of nystagmus evoked by the supine roll test in patients with horizontal semicircular canal canalolithiasis (HSC-Can) were analyzed, according to Ewald’s first law. It provided a basis for the study of human horizontal semicircular canal function and structure, objective diagnosis, and treatment of benign paroxysmal positional vertigo (BPPV).

**Materials and methods:**

The records of patients that had been tested with 2-dimensional videonystagmography (2D-VNG) were reviewed between June 2019 and June 2021. The intensity and direction of horizontal and vertical nystagmus elicited by the supine roll test were analyzed in 189 patients with idiopathic HSC-Can.

**Results:**

All the 189 patients with HSC-Can were induced horizontal nystagmus with the same direction as head-turning (geotropic) in the supine roll test, of which 119 patients (63.96%) had a weak vertical upward component of nystagmus on the affected and unaffected sides, 57 patients (30.16%) had a vertical downward component of nystagmus on the affected side and/or the unaffected side, and 13 patients (6.88%) had no vertical component of nystagmus on both the sides. The intensity values of the horizontal component on the affected and unaffected sides were 42.14 ± 24.78 (range: 6.26–138.00°/s) and 17.48 ± 10.91°/s (range: 2.40–53.83°/s), with a ratio of 2.96 ± 2.17:1, representing a significant difference (*p* < 0.001). We analyzed the characteristics of horizontal and vertical components in 119 patients with HSC-Can (57 L-HSC-Can and 62 R-HSC-Can) on the supine roll test. The intensity values of the horizontal component on the affected and unaffected sides were 43.17 ± 23.76 (range: 8.60–124.51°/s) and 17.98 ± 10.99°/s (range: 2.40–53.83°/s), and the intensity values of the vertical component on the affected and unaffected sides were 10.65 ± 8.46 (range: 1.90–50.83°/s) and 4.81 ± 3.45°/s (range: 0.30–20.43°/s), representing a significant difference between groups (*p* < 0.001). Among 119 patients with HSC-Can who had a vertical upward component on both the affected and unaffected sides in the supine roll test, unilateral weakness (UW) was normal in 53 cases and abnormal in 51 cases, and 15 cases did not undergo the caloric test. We compared the horizontal and vertical components of nystagmus induced on the affected and unaffected sides in the supine roll test in 53 patients with normal UW and 51 patients with abnormal UW, and the difference was not statistically significant.

**Conclusion:**

There is mostly a vertical upward component based on the horizontal component in HSC-Can, and the direction and intensity characteristics of nystagmus accord with Ewald’s first law, which can provide a basis for the study of human HSC function and structure, objective diagnosis, and treatment of BPPV.

## Introduction

Benign paroxysmal positional vertigo (BPPV), the most common perivestibular disease, has gained consensus on the pathogenesis of canalolithiasis and cupulolithiasis (von Brevern et al., 2015; Kim et al., 2021). Based on this theory and Ewald’s law, the diagnostic guidelines for BPPV and various repositioning therapy methods have achieved good clinical diagnosis and treatment effects for most patients (Lou et al., 2020; Power et al., 2020; Abdulovski and Klokker, 2021). The research on the characteristics and mechanism of nystagmus also has attracted attention (Chen et al., 2013; Jafarov et al., 2020; Yu et al., 2021). Previous studies on horizontal semicircular canal canalolithiasis (HSC-Can) nystagmus mainly discussed the characteristics of horizontal nystagmus in the supine roll test (Vats, 2021; Zhang et al., 2021). According to Ewald’s first law, the plane of the eye movement is the same as that of the stimulated semicircular canal. Therefore, HSC-Can nystagmus is not simple horizontal nystagmus, but positional nystagmus with horizontal components and weak vertical components. However, there are still few studies on the horizontal and vertical components of HSC-Can nystagmus and their relationship. The purpose of this study was to record and analyze the direction and intensity characteristics of the horizontal and vertical components of nystagmus induced by the supine roll test in patients with HSC-Can by using 2-dimensional videonystagmography (2D-VNG) and to further explore its mechanism, to provide a basis for the study of human HSC function and structure, objective diagnosis, and treatment of BPPV.

## Materials and methods

### Subjects

This prospective study involved the assessment of 189 patients with HSC-Can, examined at the Ear, Nose, and Throat (ENT) Department of MY Hospital, Tianjin First Central Hospital, between June 2019 and June 2021. Of 189 patients, 83 patients had L-HSC-Can, and 106 patients had R-HSC-Can. All the subjects provided informed consent before their inclusion in the study. The study procedures were approved by the Ethics Committee of the Tianjin First Central Hospital.

#### Inclusion criteria

(1) Patients with a history of positional vertigo as a predominant symptom, or associated with other complaints, such as dizziness, nausea, and vomiting.

(2) Patients diagnosed with geotropic HSC-Can, according to the benign paroxysmal positional vertigo: Diagnostic criteria (Brevern et al., 2015).

(3) No abnormalities were found in brain CT and MRI.

#### Exclusion criteria

(1) Patients with apogeotropic HSC-Can, superior semicircular canal canalolithiasis (SSC-Can), posterior semicircular canal canalolithiasis (PSC-Can), multiple canal canalolithiasis, cupulolithiasis, spontaneous, or other types of positional nystagmus.

(2) Patients with Meniere’s disease (MD), vestibular neuritis (VN), sudden deafness (SD), Ramsay Hunt syndrome, labyrinthitis, or other peripheral diseases.

(3) Patients with head trauma, vestibular migraine, stroke, and other central vestibular vertigo and balance disorders.

## Materials and methods

We obtained a detailed medical history, focusing primarily on the type of vertigo, including the onset of symptoms, and their severity, duration, and associated factors. Induction of nystagmus and corresponding parameters were observed and recorded using 2-dimensional videonystagmography (2D-VNG; France Synapsys) in the supine roll tests. The supine roll test was used to diagnose HSC-Can and consisted of turning the head from the supine to lateral position (left or right) when the patient was lying down in a supine position, with the head maintained at a 30° upward angle. The procedure was undertaken in line with the clinical practice guidelines (Bhattacharyya et al., 2017).

The direction and intensity of nystagmus, including horizontal and vertical components, were recorded in the left and right head positions on the supine roll test. According to Synapsys parameters, left and right nystagmus are considered horizontal phases, while the fast phase upward was defined as right nystagmus, and the fast phase downward was defined as left nystagmus. Vertical nystagmus includes upward and downward nystagmus, and the fast phase upward was defined as upbeat nystagmus, and the fast phase downward was defined as downbeat nystagmus. The peak slow-phase velocity within 5 s from the onset of nystagmus was recorded as the intensity of nystagmus.

In 189 patients with HSC-Can after the supine roll test, 159 of them underwent the caloric test, the other 30 cases did not undergo the caloric test. The unilateral weakness (UW) ≥ 25% is abnormal.

### Analysis

We compared the parameters of horizontal and vertical components of nystagmus elicited by the supine roll test. IBM SPSS Statistics 22 (IBM SPSS, Turkey) and JASP 0.16.3 (JASP, Netherlands) were used for statistical analyses. The quantitative data are presented as mean ± SD values and plotted using GraphPad Prism version 5 (GraphPad, San Diego, California, United States). Statistically significant differences were determined by an unpaired Student’s *t*-test. A value of *P* < 0.05 and Cohen’s *d* ≥ 0.8 were considered statistically significant.

## Results

### General demographic characteristics of subjects

Patients with HSC-Can (45 men and 144 women) ranged from 18 to 83 years (mean 53.92 years). Of these, 83 patients with left HSC-Can (20 men and 63 women) ranged in age from 26 to 74 years (mean 53.20 years), and 106 patients with right HSC-Can (25 men and 81 women) ranged in age from 18 to 83 years (mean 54.48 years). Demographic data for left HSC-Can and right HSC-Can are shown in Table 1. There were no significant differences in age or sex ratio between the two groups (*p* > 0.05).

**TABLE 1:** Demographic features of subjects in the L- and R-HSC-Can groups.

Group feature	L-HSC-Can	R-HSC-Can	HSC-Can
Number	83	106	189
Age (years)*	53.20 ± 12.58	54.48 ± 13.81	53.92 ± 13.26
Sex (M: F)*	20 : 63	25 : 81	45 : 144

HSC-Can, horizontal semicircular canal canalolithiasis; L-HSC-Can, Left HSC-Can; R-HSC-Can, Right HSC-Can; M, male; F, female; *p > 0.05.

### Horizontal and vertical components of nystagmus evoked by the supine roll test in patients with horizontal semicircular canal canalolithiasis

**FIGURE 1:**
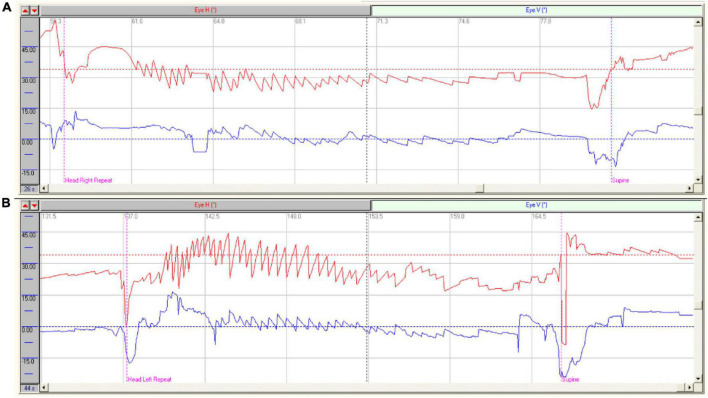
Characteristics of nystagmus in patients with HSC-Can. **(A)** Characteristics of nystagmus in a patient with R-HSC-Can: Right horizontal positional nystagmus (37.90°/s, red), accompanied by a weak vertical upward nystagmus (10.10°/s, blue) on the head-right position; **(B)** Characteristics of nystagmus in a patient with L-HSC-Can: Left horizontal positional nystagmus (39.80°/s, red), accompanied by a weak vertical upward nystagmus (10.30°/s, blue) on the head-left position.

**FIGURE 2:**
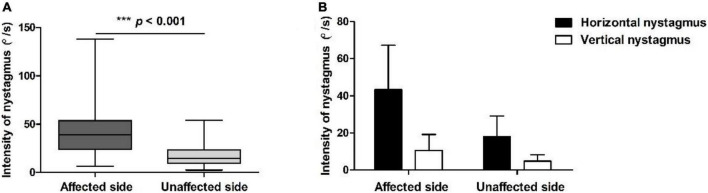
Positional nystagmus on the affected and unaffected sides in patients with HSC-Can. **(A)** The intensity values of horizontal nystagmus in 189 patients with HSC-Can on the affected and unaffected sides were 42.14 ± 24.78 (range: 6.26–138.00°/s) and 17.48 ± 10.91°/s (range: 2.40–53.83°/s), with a ratio of 2.96 ± 2.17:1, respectively (*p* < 0.001, Cohen’s *d* = 1.228). **(B)** The horizontal and vertical nystagmus in 119 patients with HSC-Can on the supine roll test. The intensity values of horizontal nystagmus on the affected and unaffected sides were 43.17 ± 23.76 (range: 8.60–124.51°/s) and 17.98 ± 10.99°/s (range: 2.40–53.83°/s), and the intensity values of vertical nystagmus on the affected and unaffected sides were 10.65 ± 8.46 (range: 1.90–50.83°/s) and 4.81 ± 3.45°/s (range: 0.30–20.43°/s), representing a significant difference between groups (*p* < 0.001, Cohen’s *d* ≥ 0.8).

All the 189 patients with HSC-Can were induced horizontal nystagmus with the same direction as head-turning (geotropic) in the supine roll test, of which 119 patients (63.96%) had a weak vertical upward nystagmus (Figure 1), 57 patients (30.16%) had vertical downward nystagmus on the affected side and/or the unaffected side, and 13 patients (6.88%) had no vertical nystagmus on both the sides. The intensity values of horizontal nystagmus on the affected and unaffected sides were 42.14 ± 24.78 (range: 6.26–138.00°/s) and 17.48 ± 10.91°/s (range: 2.40–53.83°/s), with a ratio of 2.96 ± 2.17:1, representing a significant difference (*p* < 0.001, Cohen’s *d* = 1.228; Figure 2A). This is consistent with our previous results (Zhang et al., 2021).

This study induced a weak vertical upward nystagmus in 119 of 189 (62.96%) patients with HSC-Can on the affected and unaffected sides in the supine roll test. A total of 57 patients (30.16%) had vertical downward nystagmus on the affected side and/or the unaffected side, among which, 12 cases had a weak vertical downward nystagmus on both the sides, 32 cases had vertical upward nystagmus on one side and vertical downward nystagmus on the other side, 13 cases had vertical nystagmus on one side and no vertical nystagmus on the other side, and the rest 13 cases had no vertical nystagmus on both the sides (Table 2).

**TABLE 2:** The vertical component of nystagmus evoked by the supine roll test on the affected and unaffected sides in 189 patients with HSC-Can.

Head position		Affected side		
	Vertical component	Up (cases,%)	Down (cases,%)	No (cases,%)
Unaffected side	*Up (cases,%)*	119/189, (62.96%)	16/189, (8.47%)	2/189, (1.06%)
	*Down (cases,%)*	16/189, (8.47%)	12/189, (6.34%)	2/189, (1.06%)
	*No (cases,%)*	6/189, (3.17%)	3/189, (1.59%)	13/189, (6.88%)

Up, vertical upward nystagmus; Down, vertical downward nystagmus, No, no vertical nystagmus.

Considering that the cause of vertical downward nystagmus is unknown, we excluded 57 patients with HSC-Can with vertical downward nystagmus and 13 patients with HSC-Can with no vertical nystagmus, and only analyzed the characteristics of horizontal and vertical nystagmus in 119 patients with HSC-Can (57 L-HSC-Can and 62 R-HSC-Can) on the supine roll test (Figure 2B). The intensity values of horizontal nystagmus on the affected and unaffected sides were 43.17 ± 23.76 (range: 8.60–124.51°/s) and 17.98 ± 10.99°/s (range: 2.40–53.83°/s), and the intensity values of vertical nystagmus on the affected and unaffected sides were 10.65 ± 8.46 (range: 1.90–50.83°/s) and 4.81 ± 3.45°/s (range: 0.30–20.43°/s), representing a significant difference between groups (*p* < 0.001, Cohen’s *d* ≥ 0.8; Table 3).

**TABLE 3:** Comparisons of horizontal and vertical nystagmus evoked by the supine roll test on the affected and unaffected sides in 119 patients with HSC-Can.

	Horizontal (°/s)	Vertical (°/s)	*t*-value	*P*-value	Cohen’s *d* (95%CI)
Affected side	43.17 ± 23.76	10.65 ± 8.46	17.89	<0.001	1.626
Unaffected side	17.98 ± 10.99	4.81 ± 3.45	15.66	<0.001	1.423
*t*-value	14.24	6.92			
*p*-value	<0.001	<0.001			
Cohen’s *d* (95%CI)	1.294	0.804			

### Nystagmus of horizontal semicircular canal canalolithiasis and the function of horizontal semicircular canal

Among 189 patients with HSC-Can, 159 cases completed the caloric test, including 82 cases with normal UW and 77 cases with abnormal UW, and 30 cases did not undergo the caloric test. In the 119 patients with HSC-Can who had a vertical upward component of nystagmus on the affected and unaffected sides in the supine roll test, 104 patients of them underwent the caloric test, UW was abnormal in 53 cases and normal in 51 cases, and the other 9 cases did not undergo the caloric test. There were no statistically significant differences in horizontal and vertical components of nystagmus between normal and abnormal UW cases on the affected and unaffected sides in the supine roll test (Table 4).

**TABLE 4:** Comparisons of horizontal and vertical nystagmus evoked by the supine roll test on the affected and unaffected sides in patients with HSC-Can with normal and abnormal UW.

		Abnormal UW (51 cases)	Normal UW (53 cases)	t-value	*P*-value
Affected side	H (°/s)	46.41 ± 24.96	41.46 ± 22.83	1.06	0.29
	V (°/s)	11.85 ± 9.98	9.50 ± 6.18	1.45	0.15
Unaffected side	H (°/s)	18.46 ± 12.36	16.92 ± 9.98	0.70	0.48
	V (°/s)	5.12 ± 3.78	4.71 ± 3.51	0.57	0.57

UW, unilateral weakness; H, the intensity of horizontal component; V, the intensity of vertical component.

## Discussion

Multiple semicircular canals are involved in unilateral peripheral vestibular diseases (a single semicircular canal is rare), and the spontaneous nystagmus resulting is mostly horizontal or torsional. The relationship between the direction of spontaneous nystagmus and the injured semicircular canal is not completely clear. The reason that there is still a lack of vestibular detection technology to fully evaluate each semicircular canal. Nystagmus of HSC-Can, a single factor stimulus response induced from a single HSC, has the physiological properties of a single HSC. It is also the best way to analyze the characteristics of HSC nystagmus through HSC-Can based on Ewald’s laws (Zhang et al., 2021).

We analyzed the characteristics of horizontal and vertical components of nystagmus evoked by the supine roll test in 189 patients with HSC-Can, and found that there was mostly a vertical upward component based on the horizontal component in HSC-Can. The direction and intensity characteristics of HSC-Can nystagmus accord with Ewald’s first law and the anatomical characteristics of HSC at about 30° from the horizontal plane. Moreover, there was no significant difference in horizontal and vertical components of nystagmus between normal and abnormal UW subjects in affected- and unaffected-head position of the supine roll test, which further suggests that the nystagmus of HSC-Can can reflect the anatomical and physiological characteristics of HSC and provide a basis for the study of HSC function, objective diagnosis, and treatment of BPPV.

With the in-depth study of BPPV and the application of VNG, the diagnosis and treatment of BPPV have gradually developed from subjective analysis to objective positioning (Zhu et al., 2021). Previous studies on HSC-Can nystagmus mostly focused on the analysis of horizontal nystagmus (Vats, 2021; Zhang et al., 2021). The direction and intensity of horizontal nystagmus induced by the supine roll test were measured by subjective visual measurement or objective quantitative method. Based on VNG technology, the development from single horizontal nystagmus to two-dimensional nystagmus will be also one of the research focus of HSC-Can nystagmus. Further exploring the characteristics of horizontal and vertical components of HSC-Can nystagmus will help to deeply understand the physiological characteristics of human HSC and provide a basis for clinical research of HSC-Can.

The caloric test, a routine vestibular function test, detects the low-frequency vestibulo-ocular reflex (VOR) function of the HSC through different temperature stimulations, which can induce horizontal nystagmus with or without weak vertical nystagmus. The vertical nystagmus induced in caloric testing may be due to the local stimulation of temperature that affects multiple semicircular canals ipsilaterally (anterior, horizontal, and posterior semicircular canals), and may also be related to the presence of central lesions in the patient (Chen et al., 2007). In addition, research has shown the simultaneous occurrence of a non-thermoconvective mechanism, which theoretically could answer for about one-third of the total caloric response. It has been assumed that this latter mechanism underlies the caloric nystagmus induced in microgravity (Stahle, 1990). Therefore, it is not suitable to analyze the anatomical angle and gravity relationship of HSC through the characteristics of nystagmus induced by caloric test. Otoconia are only a single factor stimulation to HSC-Can-induced nystagmus, and are completely derived from the gravity effect. At present, any other clinical vestibular test method is difficult to simulate, and simple unilateral HSC damage is rare in clinical methods. Therefore, HSC-Can is an excellent physiological stimulation model of HSC in humans.

In HSC-Can, gravity induces the rolling of the otoconia from the posterior arm of the HSC toward the ampulla on the affected side, driving the endolymph to the ampulla, while the otoconia roll to the canal from the ampulla driving the endolymph away from the ampulla (Martellucci et al., 2020; Zhang et al., 2020). This results in a single stimulus to the HSC without involving the anterior and posterior semicircular canals. According to Ewald’s first law, the plane of eye movement in the aVOR is the same as that of the stimulated semicircular canal. The nystagmus in patients with HSC-Can has horizontal and vertical components, and we speculate that this may be caused by the angle between the HSC and the horizontal plane.

A total of 189 patients with HSC-Can were included in this study, of which 119 patients (62.96%) were induced with left/right horizontal nystagmus (geotropic nystagmus), accompanilane (about 30 ? above the horizontal plane).ed by a weak vertical upward nystagmus on the affected and unaffected sides in the supine roll test. The other 70 cases (37.04%) had horizontal nystagmus consistent with HSC-Can nystagmus, while the forms of vertical nystagmus were different. We suspected that 70 patients who had vertical downward nystagmus or no vertical nystagmus might have heterophoria (Barton and Ranalli, 2021), which needs to be further studied in the future. The vertical component of nystagmus in 119 cases (62.96%) was upward when the head was positioned on both the affected and unaffected sides. This finding was related to the direction of nystagmus—always toward the side with higher vestibular tension. Taking R-HSC-Can as an example, when the patient moved from supine to right head position, the otoconia of the right HSC rolled toward the ampulla, resulting in excitatory stimulation. Right horizontal nystagmus accompanied by a vertical upward component can be induced (Figure 1A), and its direction was consistent with the HSC plane (about 30° above the horizontal plane). Therefore, the supine roll test induced horizontal nystagmus accompanied by a weak vertical upward component. When the patient turned to the left head position, the otoconia of the right HSC rolled away from the ampulla to produce inhibitory stimulation, and the tension of the left HSC increased. A weak left horizontal nystagmus accompanied by a vertical upward component can be induced (Figure 1B), and its direction was consistent with the left HSC plane (about 30° above the horizontal plane).

In the other 70 cases (37.04%), the horizontal component of nystagmus accorded with the characteristics of HSC-Can nystagmus. However, the vertical component had different forms, including several different situations: vertical downward nystagmus on the affected side and vertical upward nystagmus on the unaffected side; vertical upward nystagmus on the affected side and vertical downward nystagmus on the unaffected side; vertical downward nystagmus on both the affected and unaffected sides; and no vertical nystagmus on the affected and/or unaffected side. For HSC-Can, the vertical component of nystagmus induced by otoconia rolling is correlated with the spatial orientation of the horizontal semicircular canal, and there is no otoconia stimulation of the posterior semicircular canal. Nystagmus of HSC-Can is a comprehensive vector of the horizontal component and vertical upward component. When the direction and intensity of the vertical component change, it is speculated that the patients with HSC-Can may have other semicircular canal or otolithic lesions, or be related to the patients’ heterophoria (Barton and Ranalli, 2021).

Our previous study showed that HSC-Can could demonstrate Ewald’s law in the human body and be used as a physiological stimulus model to obtain a deeper understanding of the characteristics of the human HSC (Zhang et al., 2021). The intensity and direction of HSC-Can nystagmus are mainly related to the stimulation effect of the rolling otoconia induced by gravity. Nystagmus is based on the functional existence of the involved HSC, and may also be affected by other factors, such as the function of ASC, PSC, or otolith organ. Our study showed that among 119 patients with HSC-Can, 51 patients had abnormal UW and 53 patients had normal UW, which were similar to previous reports (Bi et al., 2019). However, there was no significant difference in horizontal and vertical components of nystagmus between normal and abnormal UW subjects in affected- and unaffected-head position of the supine roll test, which further suggests that the nystagmus of HSC-Can can reflect the anatomical and physiological characteristics of HSC.

### Questions to be studied

Following the BPPV diagnostic criteria, a systematic vestibular examination was not carried out for patients with HSC-Can, such as vestibular evoked myogenic potential (VEMP) and multifrequency tests of three semicircular canals. The influence from otoliths lesion and other frequency lesions of the three semicircular canals for nystagmus of HSC-Can cannot be excluded. Therefore, the reason why the vertical component of nystagmus was not upward in 70 of 189 cases cannot be clearly explained yet. On the contrary, the presence of a vertical downward component, or the nystagmus does not conform to Ewald’s first law in HSC-Can that should be further studied whether is revealed HSC-Can combined with other semicircular canals or otolith organ lesions.

## Conclusion

In this study, the direction and intensity characteristics of horizontal and vertical components of HSC-Can nystagmus evoked by the supine roll test were recorded and analyzed. The results showed that most of them had vertical upward components except horizontal components, suggesting that HSC-Can has the characteristics of the human HSC physiological effect model. The nystagmus follows Ewald’s first law, which can provide a basis for the study of HSC function and objective diagnosis and treatment of HSC-Can and semicircular canal-related diseases.

## Data availability statement

The raw data supporting the conclusions of this article will be made available by the authors, without undue reservation.

## Ethics statement

The studies involving human participants were reviewed and approved by this study was approved by the Institutional Review Board of Tianjin First Central Hospital (Tianjin, China). The patients/participants provided their written informed consent to participate in this study.

## Author contributions

WW and TC performed the study design. XZ, QD, QL, and CW acquired and analyzed the data. XZ and TC drafted the manuscript. All authors data interpretation and critical revision of the manuscript.
